# Yeast communities related to honeybees: occurrence and distribution in flowers, gut mycobiota, and bee products

**DOI:** 10.1007/s00253-023-12942-1

**Published:** 2024-01-26

**Authors:** Alice Agarbati, Silvia Gattucci, Laura Canonico, Maurizio Ciani, Francesca Comitini

**Affiliations:** Department of Life and Environmental Sciences, Polytechnic University of Marche, Via Brecce Bianche, 60131 Ancona, Italy

**Keywords:** Honeybees, Honeybees gut, Yeasts detection, Bee pollen, Bee bread, Propolis

## Abstract

**Abstract:**

Honeybee (*Apis mellifera*) is an important agricultural pollinator and a model for sociality. In this study, a deep knowledge on yeast community characterizing the honeybees’ environmental was carried out. For this, a total of 93 samples were collected: flowers as food sources, bee gut mycobiota, and bee products (bee pollen, bee bread, propolis), and processed using culture-dependent techniques and a molecular approach for identification. The occurrence of yeast populations was quantitatively similar among flowers, bee gut mycobiota, and bee products. Overall, 27 genera and 51 species were identified. *Basidiomycetes* genera were predominant in the flowers while the yeast genera detected in all environments were *Aureobasidium*, *Filobasidium*, *Meyerozyma*, and *Metschnikowia*. Fermenting species belonging to the genera *Debaryomyces*, *Saccharomyces*, *Starmerella*, *Pichia*, and *Lachancea* occurred mainly in the gut, while most of the identified species of bee products were not found in the gut mycobiota. Five yeast species, *Meyerozyma guilliermondii*, *Debaryomyces hansenii*, *Hanseniaspora uvarum*, *Hanseniaspora guilliermondii*, and *Starmerella roseus*, were present in both summer and winter, thus indicating them as stable components of bee mycobiota. These findings can help understand the yeast community as a component of the bee gut microbiota and its relationship with related environments, since mycobiota characterization was still less unexplored. In addition, the gut microbiota, affecting the nutrition, endocrine signaling, immune function, and pathogen resistance of honeybees, represents a useful tool for its health evaluation and could be a possible source of functional yeasts.

**Key points:**

• *The stable yeast populations are represented by M. guilliermondii, D. hansenii, H. uvarum, H. guilliermondii, and S. roseus.*

• *A. pullulans was the most abondance yeast detective in the flowers and honeybee guts.*

• *Aureobasidium, Meyerozyma, Pichia, and Hanseniaspora are the main genera resident in gut tract.*

**Supplementary Information:**

The online version contains supplementary material available at 10.1007/s00253-023-12942-1.

## Introduction

Honeybees (*Apis mellifera*) belong to the family *Apidae*, order Hymenoptera, and are insects with a central role in many biological and economic aspects (Eimanifar et al. 2018). They produce not only honey and other virtuous bee products largely utilized by humans (Anjum et al. 2019), but they have an important effect on ecosystem. Indeed, they increase productivity and profitability in the agricultural sector (Hroncova et al. 2019) and contribute to maintaining biodiversity through the pollination of numerous plants (Eimanifar et al. 2018).

Honeybees are organized in hives, in which they are genetically related and work as collective unit but include both single and group interactions between hive components (Berenbaum and Liao, 2019; Lamei et al. 2020). The hive system undergoes continuous exchange with its surroundings, in which biotic and abiotic interactions crucially influence the bees’ microbiota composition (Hughes et al. 2008; Khan et al. 2020). The honeybee is a social insect that lives in large perennial communities in direct contact with a dynamic ecosystem and has developed a strong and massive microbiota over time that supports the insect in every stage of its development (Zheng et al. 2018). It is responsible for multiple functions similar to those of the human intestinal microbiome, such as positive involvement in energy metabolism for the homeostasis of sugars and lipids (Romero et al. 2019). Furthermore, the microorganisms that constitute the stable portion of the microbiota participate in the production of secondary metabolites essential for bees, such as vitamins and short-chain fatty acids, with a consequent variation in the chemical and physical conditions of the life environment. Finally, the microbiota actively participates in the stimulation of the immune system and prevents the onset of gastrointestinal pathologies through direct competition (El Khoury et al. 2018; Motta et al. 2020).

Many researchers in the last decade have developed an interest in the role of the gastrointestinal microbiome of the bee and investigated the specific microbial components that constitute the gut microbiome. There are five ubiquitous bacterial phyla that are always present within the gastrointestinal system (Tola et al. 2020). These are (i) *Snodgrassella alvi* belonging to the *Neisseriaceae* family, with a crucial role in the formation of the biofilm in the intestinal walls, (ii) the gamma proteobacterium *Gilliamella apicola* belonging to the *Orbaceae* family that is able to ferment sugars, (iii) *Lactobacillus* firm-4, (iv) *Lactobacillus* firm-5, and (v) the actinobacterium *Bifidobacterium asteroides*. At lower concentrations, four rare members are also present, represented by the proteobacteria *Frischella perrara*, *Bartonella apis*, *Bombella apis*, *Commensalibacter*, and *Apibacter* (Ludvigsen et al., 2020).

Within the gastrointestinal tract of the bee, there is also a eukaryotic portion represented by yeast species (Vega and Dowd 2005; Zheng et al., 2019) . This presence is undoubtedly attributable to the enormous dispersion of yeasts in nature since they are considered ubiquitous microorganisms frequently found in soil and the air and on vegetable surfaces, animals and foods, mostly highly sugary substrates such as flower nectar and fruit juices (Herrera et al. 2009; Chappell and Fukami 2018).

The eukaryotic portion of the bee microbiota, called mycobiota, although present in lower concentrations compared to bacteria, has the same colonization behavior with a progressive increase starting from the colon up to the rectum. A study by Callegari et al. (2021) carried out on 3 different subspecies of bees (*A. mellifera ligustica*, *A. mellifera jemenitica*, and *Apis florea*) showed that the most abundant genus of mycobiota is represented by *Starmerella* spp. (31%), followed by *Hanseniaspora* spp. (12%), *Aspergillus* spp., and *Naganisha* spp. (both 11%), and the genera *Aureobasidium* sp., *Moniniella* sp., *Candida* sp., and *Penicillium* sp. were found in lower percentages.

The present research aims to characterize the microbial communities of honeybees consisting of three families of *A. mellifera* subspecies *ligustica*, with the aim to determine some correlations among yeasts honeybees, their products and environments, also considering the seasonality. The gut tract of the bees, wildflowers within an area of 5 km (taking into account the daily travel distances), and bee products such as pollen, bee bread, and propolis were sampled during 2021 considering the seasonality (spring–summer and winter). After the evaluation of the total viable microorganisms, the yeast community was mapped, and the seasonal ecological distribution was observed to establish the relationships between insects and the environment as well as the potential role of yeasts.

## Methods

### The origin of the hives and the relative bees

Three hives (named C, M, and U) of honeybees (*A. mellifera ligustica*, *Hymenoptera: Apidae*) and the related surrounding environment within 3–5 km were used as sources of samples. Hives were located in the Fabriano locality (Marche region, central Italy) (43°20′18.82″N and 12°54′30.67″W; 325 m a.s.l), and the sampling process was carried out seasonally in spring–summer (May–June-July–August-September 2021) and winter (October–November-December 2021). During each period, sampling was performed once a week.

### Collection and processing of samples

During the full isolation strategy, a total of 93 samples, listed in Table 1, were collected and grouped into three typologies: (i) bee food supply; (ii) gut tract of forager bees; and (iii) bee products such as corbicula pollen, propolis, and bee bread. The bee food supply environment was represented by different species of flowers as sources of nectar for bees that are commonly used between hives. In particular, 24 samples of entire flowers (that were in season, as specified in Table 1) belonging to 23 different species were collected, under sterile conditions as listed in Table 1. In particular, each flower (typology i) was cut with sterilized scissors and putted inside a sterile tube containing 9 ml of sterile water, by using sterile tweezers. In the laboratory, the tubes were shaken for 5 min at 120 rpm/min and the liquid sample was processed for viable cell counts. For sampling of bees gut (typology ii), the following modality was applied: the bees were put on cold chamber (4 °C) overnight to induce chill-coma before the dissection. The gut was dissected out in sterile conditions by pulling out the stinger. The gut was suspended in 500 μl of PBS buffer and glass balls were added to constitute about 25% of the final volume after extraction. The mechanical disruption of the gut walls was achieved by subjecting them to 1 min of vertexing at 9000 rpm, followed by an additional minute on ice. Finally for bee products (typology iii), a empiric amount of propolis, bee pollen, or bee bred was collected separately by using sterile tools (tweezers, bistoury, and collector straw). In the laboratory, the harvested samples were weighed and serial dilution in sterile water was carried out.

**Table 1:**
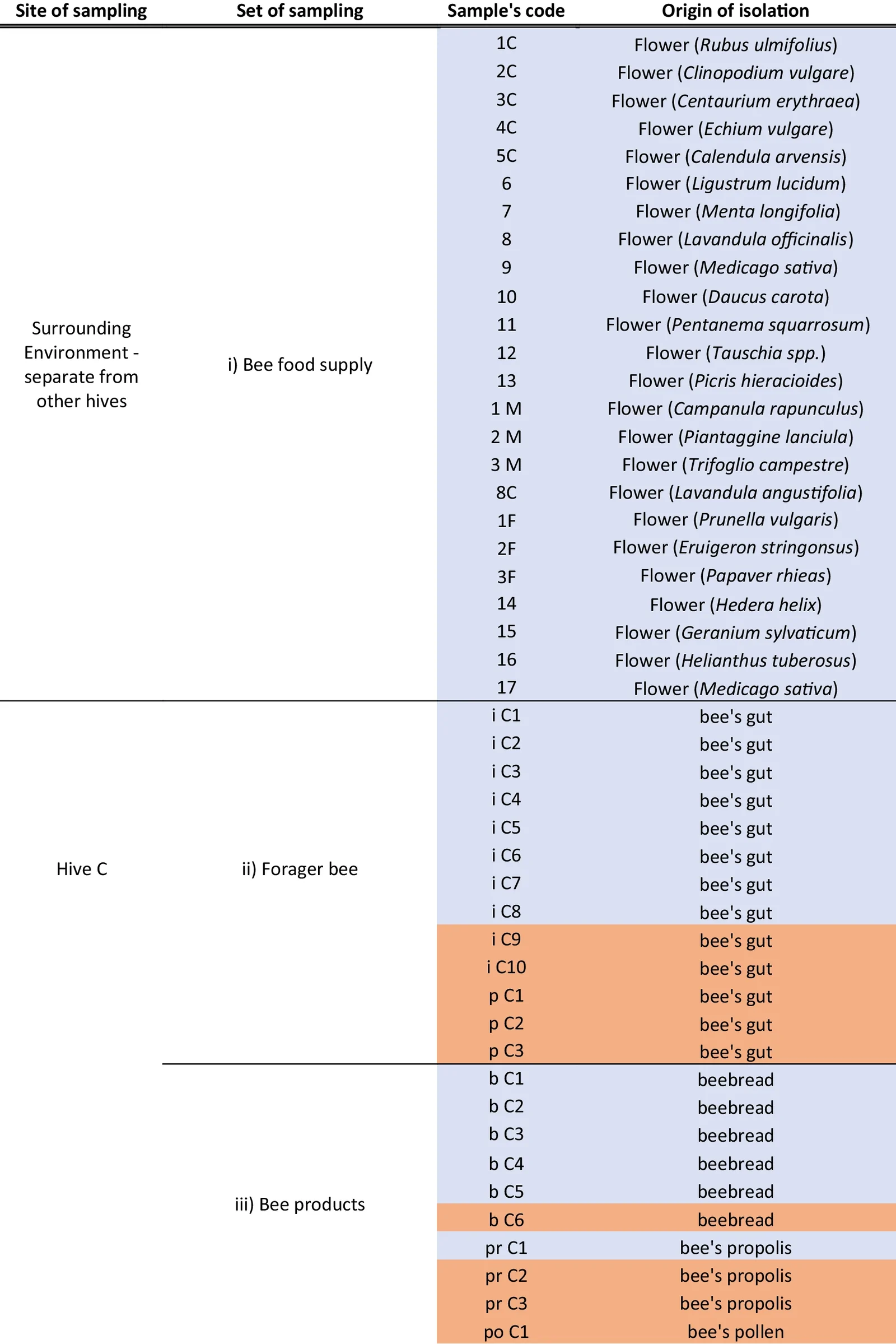

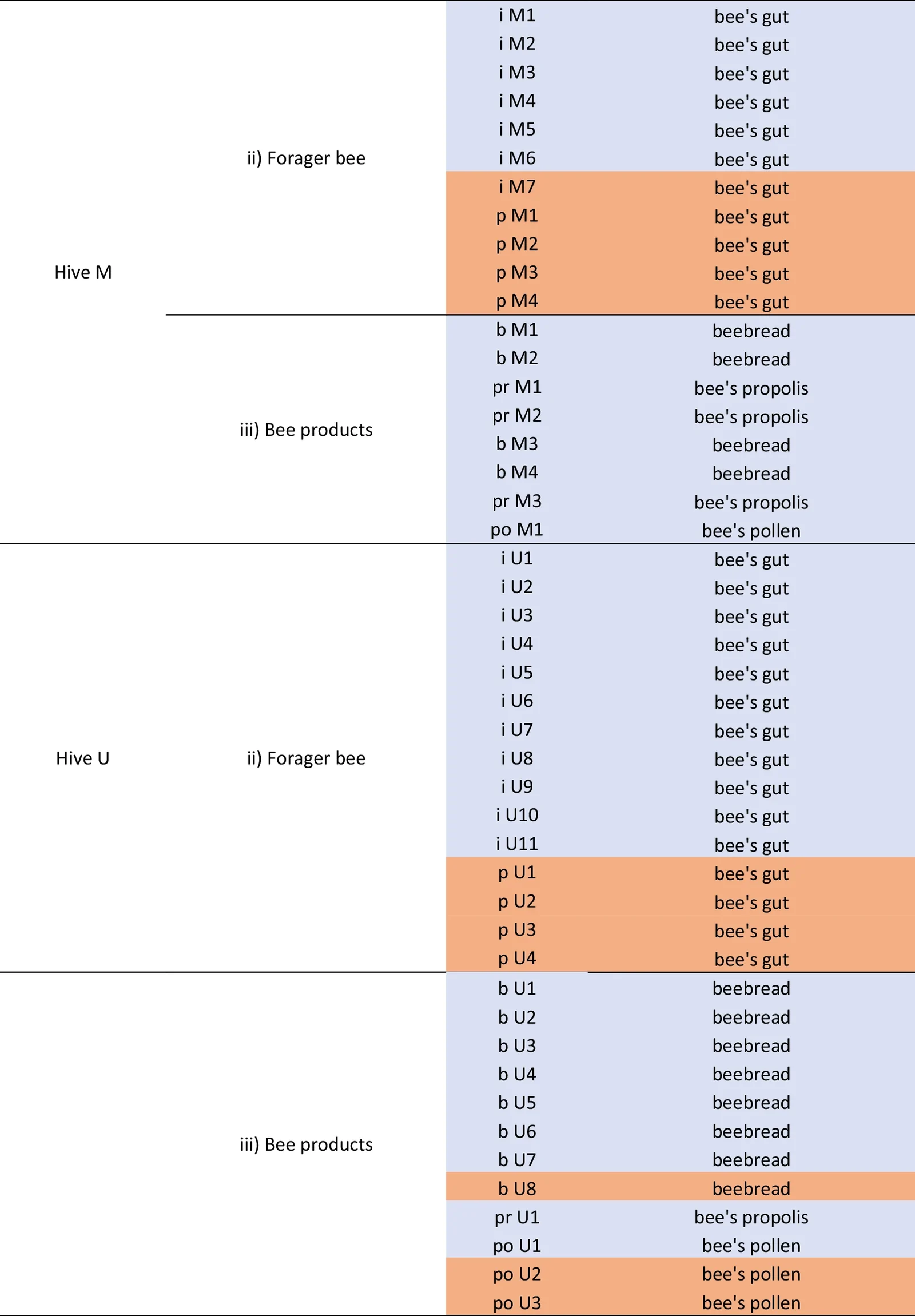
Source and origin of samples. Season when sampled: summer (May-June-July August-September), colored blue; winter (October-November-December), colored red

In each hive (C, M, U), 23, 19, and 27 samples were collected, respectively (Table 1). Regarding hive C, 13 samples belonged to the (ii) typology, and 10 samples belonged to the (iii) typology (of which 6 samples were bee bread, 3 were bee propolis, and 1 was pollen). Regarding hive M, 11 samples belonged to the (ii) typology, and 8 samples belonged to the (iii) typology (of which 4 samples were bee bread, 3 were bee propolis, and 1 was pollen). Finally, regarding the U hive, 15 samples belonged to the (ii) typology, and 12 samples belonged to the (iii) typology (of which 8 samples were bee bread, 1 was bee propolis, and 3 were pollen). Moreover, the season in which the sample was collected is specified in Table 1.

All samples were collected aseptically using sterile plastic bags, preserved by refrigeration and processed in the laboratory multiple times.

Each sample was subjected to decimal serial dilutions in 0.9% sterile peptone water, homogenized for 1 min, and spread on WL nutrient agar plates (Wallerstein Laboratories, Oxoid, Hampshire, UK) and Rose Bengal Base Agar (Oxoid, Hampshire, UK) supplemented with 0.005% chloramphenicol (Sigma‒Aldrich, Saint Louis, MO, USA) to suppress bacterial growth. WL nutrient agar and Rose Bengal Base Agar plates were incubated at 25 °C for yeast and mold detection, respectively. The microbial population was expressed as colony-forming unit (CFU) per gut sample, CFU/flower, and CFU/g of bee product for their corresponding samples.

### Yeast occurrence, isolation, and molecular identification

Yeast isolation, after macro- and micromorphological analysis, was performed in proportion to the frequency of each yeast morphotype by plates containing between 30 and 300 colonies. Approximately 10% of the colonies per plate were purified on YPD agar plates (1% yeast extract, 2% glucose, 2% peptone, 2% agar) and cryopreserved for a long time at − 80 °C in YPD broth containing 40% glycerol. Purified yeast morphotypes were then molecularly identified.

The DNA of 117 purified isolates was extracted according to the method described by Stringini et al. (2008). The ITS1-5.8S rRNA-ITS2 region was amplified by PCR for yeast identification following the procedure reported by Agarbati et al. (2019). Briefly, the primer set ITS1 (5′-TCCGTAGGTGAACCTCGCG-3′) and ITS4 (5′-TCCTCCGCTTTATTGATATGC-3′) was used, and the PCR products were separated by horizontal electrophoresis (Bio-Rad, Hercules, CA, USA) in a 1.5% (w/v) agarose gel using 0.5 × TBE buffer. Each yeast isolate was then identified by sequencing. All the genomic sequences obtained were compared with those already present in the data library using the BLAST program (Altschul et al. 1997) and the GenBank database. A total of 105 sequences were included in the NCBI GenBank data library under accession numbers OQ357433 to OQ357537.

### Statistical analyses

The experimental data of viable cell counts were analyzed through analysis of variance using the software STATISTICA 7, one-way ANOVA program (Statsoft Inc., Tulsa, OK, USA). The data were considered significantly different according to Duncan’s test, with an associated *p* value < 0.05.

The yeast diversity between flowers, bee gut, and bee products was analyzed by the Shannon and diversity (*D*) indices, and the relative abundances of the species recovered for each set of samples were analyzed by principal component analysis (PCA) using JMP® 11 statistical software (Statistical discovery from SAS, New York, NY, USA).

## Results

### Microbial presence in flowers, gut bees, and bee products

A culture-dependent approach using selective media was applied to detect and quantify the microbial community associated with forager bees, flowers, and bee products in three different beehives hosting honeybees belonging to the same species, *A. mellifera ligustica*.

Within each type of sample, and therefore considering the statistical variability between them, the yeast population isolated from flowers was consistently larger than that of the aerobic bacteria, even if high variability was found (Supporting Information, Table S1). Indeed, in 4 out of 24 total samples, no yeasts were isolated, while 7 samples had a limited yeast population (c.a. log 1 CFU/flower). The remaining samples had relevant cultivable yeasts (c.a. log 5 CFU/flower or higher). A similar trend was found for molds, while aerobic bacteria were generally less abundant and completely absent in 9 samples.

The results of the evaluation of cultivable microorganisms associated with the bee gut were similar among the three hives sampled. The yeast population was quite uniform, with mean values less than log 4 CFU/gut; only one out of 39 samples did not have yeast, and 5 samples had a reduced population of yeast (ca. log 1 CFU/gut). Molds were generally absent, and aerobic bacteria were on average less numerous than yeasts (log 2 CFU/gut) and were absent in approximately one-third of the samples. Finally, the mean number of fungal colony-forming units (CFUs) isolated from the bee products was approximately log 3.5 CFU/g of product, regardless of the hive sampled, and only a few samples showed no cultivable yeasts (Supplementary Results, Table S1).

A statistical evaluation of the cultivable yeasts, molds, and aerobic bacteria occurrence is reported in Table 2, considering both the specific microbial population and the season. The yeast population was similar among the three sets of samples (flowers, bee gut, and bee products) independently from seasonality. Molds had the highest concentration in flowers, while no significant seasonal differences were observed within bee gut and bee product samples, although the concentration of mold was higher in bee products than in the bee gut. Consistent variability was observed regarding aerobic bacteria concentrations with no significant differences between samples (fair homogeneity).

**Table 2:** Presence of yeasts, mold, and aerobic bacteria in all samples, grouped by seasonality (summer/winter)

	Viable cell counts		
Samples	Yeasts	Mold	Aerobic bacteria
Flowers summer	3.22 ± 2.46^a^	2.98 ± 1.86^a^	1.76 ± 1.71^ab^
Bee gut summer	3.61 ± 1.01^a^	0.16 ± 0.55^d^	2.24 ± 1.61^a^
Bee gut winter	2.58 ± 1.67^a^	0.63 ± 0.93^ cd^	0.91 ± 1.37^ab^
Bee products summer	3.65 ± 2.27^a^	1.84 ± 2.06^ab^	2.21 ± 2.37^a^
Bee products winter	3.87 ± 2.44^a^	1.54 ± 1.93^bc^	0.61 ± 1.63^b^

The microbial population was detected by viable cell counts, and the results are expressed as log CFU/flower, log CFU/gut, and log CFU/g of product for bee food supply, forager bee gut tract, and bee products. Data are reported as the mean values ± standard deviations, and different superscript letters (^a, b, c, d^) within each column are significantly different according to Duncan’s test (*p* value < 0.05)

### Molecular identification of isolated yeasts and association with the origin

After quantitative evaluation of the culturable microbial population, a comparative analysis was carried out considering the type of samples and sampling period and taking into account each relative abundance. Out of the 202 strains, only 117 were included in the molecular identification by using the ITS region, and the results are reported in Table 3.

**Table 3:** Molecular identification (ITS analyses) of yeast genera or species detected through viable cell counts for each sample typology

Source		Code of sample	Molecular identification of yeasts (ITS region analyzed)
i) Bee food supply (flowers)		1C	*Aureobasidium pullulans*
		3C	*Cryptococcus nemorosus*
		4C	None identified
		6	None identified
		7	*Cryptococcus carnescens*
		10	Buckleyzyma salicina*
		11	*Meyerozyma caribbica*
			*Aureobasidium pullulans*
			*Candida friedrichii*
			uncultured fungus
		12	Papiliotrema laurentii*
			*Aureobasidium pullulans*
		13	Papiliotrema laurentii*
			*Metschnikowia ziziphicola*
		1F	None identified
		14	*Dothiora prunorum*
			Filobasidium wieringae*
		15	Filobasidium magnum*
			*Dothiora prunorum*
		17	Symmetrospora coprosmae*
			*Cryptococcus carnescens*
ii) Forager bee gut	Hive C	i C1	*Debaryomyces hansenii*
		i C2	*Meyerozyma guilliermondii*
			*Meyerozyma caribbica*
			*Hanseniaspora pseudoguilliermondii*
			*Hanseniaspora uvarum*
		i C3	*Hanseniaspora pseudoguilliermondii*
		i C4	*Hanseniaspora uvarum*
			*Meyerozyma guilliermondii*
		i C5	*Meyerozyma caribbica*
			*Meyerozyma guilliermondii*
		i C6	*Debaryomyces hansenii*
			*Meyerozyma guilliermondii*
		i C7	None identified
		i C9	*Hanseniaspora guilliermondii*
		i C10	*Hanseniaspora uvarum*
			*Pichia fermentans*
		p C1	*Sporobolomyces roseus*
			*Rhodotorula mucilaginosa*
		p C2	*Aureobasidium pullulans*
		p C3	*Aureobasidium pullulans*
	Hive M	i M1	*Pseudozyma flocculosa*
		i M2	*Debaryomyces hansenii*
		i M3	*Yarrowia galli*
		i M4	*Pichia kluyveri*
			*Meyerozyma caribbica*
			*Hanseniaspora pseudoguilliermondii*
		i M5	*Sporobolomyces roseus*
		i M7	*Meyerozyma guilliermondii*
		p M1	*Aureobasidium pullulans*
		p M2	*Sarocladium strictum*
		p M3	*Rhodotorula kratochvilovae*
			Cystofilobasidium macerans*
		p M4	*Starmerella magnoliae*
	Hive U	i U3	*Hanseniaspora uvarum*
			*Pichia kluyveri*
		i U4	*Clavispora* spp.
		i U5	*Pichia kudriavzevii*
			*Hanseniaspora opuntiae*
			*Lachancea kluyveri*
		i U6	*Lachancea kluyveri*
			*Meyerozyma guilliermondii*
			Filobasidium wieringae*
		i U7	*Meyerozyma caribbica*
			*Meyerozyma guilliermondii*
		i U8	*Starmerella bombicola*
		i U9	*Saccharomyces cerevisiae*
			*Pichia kluyveri*
			*Hanseniaspora guilliermondii*
		i U10	None identified
		p U1	*Aureobasidium pullulans*
		p U2	*Debaryomyces hansenii*
		p U3	*Metschnikowia pulcherrima*
			*Rhodotorula babjevae*
		p U4	*Aureobasidium pullulans*
			*Rhodotorula* spp.
iii) Bee products	Hive C	b C2	*Starmerella* spp.
		b C3	*Metschnikowia ziziphicola*
			*Starmerella magnoliae*
			*Metschnikowia pulcherrima*
			*Starmerella* spp.
			*Debaryomyces hansenii*
		b C4	*Meyerozyma guilliermondii*
		b C5	Filobasidium uniguttulatum*
			*Bullera alba*
		b C6	*Aureobasidium pullulans*
		pr C1	*Meyerozyma guilliermondii*
		pr C2	*Dothioraceae* spp.
		pr C3	*Pichia terricola*
		po C1	Cystofilobasidium macerans*
			*Sarocladium* spp.
			*Metschnikowia chrysoperlae*
			Filobasidium globisporum*
	Hive M	b M1	*Aureobasidium pullulans*
		b M2	*Starmerella bombicola*
		pr M1	*Metschnikowia ziziphicola*
		pr M2	*Metschnikowia ziziphicola aff*
		b M3	*Zygosaccharomyces rouxii*
		po M1	*Metschnikowia maroccana*
			Filobasidium wieringae*
			*Meyerozyma guilliermondii*
	Hive U	b U2	Filobasidium stepposum*
			*Debaryomyces hansenii*
			*Starmerella floricola*
		b U3	*Debaryomyces hansenii*
		b U4	*Yarrowia galli*
		b U5	*Lachancea thermotolerans*
		b U6	Filobasidium oeirense*
			*Meyerozyma guilliermondii*
		b U7	*Metschnokowia reukaufii*
		b U8	Ustilago cynodontis*
		po U1	Filobasidium oeirense*
			*Aureobasidium melanogenum*
		po U2	*Metschnikowia reukaufii*
			*Starmerella apicola*
		po U3	*Starmerella apicola*
			Naganishia bhutanensis*

^*^Yeast species belonged to *Basidiomycota* phylum

Yeast identification showed a total of 51 species ascribed to 27 genera, of which 20 belonged to *Ascomycetes* and 7 belonged to *Basidiomycetes*.

In flowers, 11 different genera of yeasts, including *Ascomycetes* and *Basidiomycetes*, were identified. More than one species was found in the genera *Cryptococcus* and *Filobasidium*. Based on this, the presence of *Aureobasidium*, *Metschnikowia*, and *Papiliotrema* is largely justified.

Seventeen different genera of yeasts were detected in the bee gut, regardless of the hive of origin. *Hanseniaspora* was the most frequent genus, with four different species, followed by *Pichia* and *Rhodotorula*, with three species each.

Of these 17 different genera and related species able to survive in the gastrointestinal tract, 4 were also found in flowers (*Aureobasidium* sp., *Filobasidium* sp., *Metschnikowia* sp., and *Meyerozyma* sp.) as evidence of a continuous flux of yeasts between flowers and bees.

Yeasts found in the gut but not isolated in flower samples were *Sporobolomyces* sp., *Clavispora* sp., *Debaryomyces* sp., *Starmerella* sp., *Hanseniaspora* sp., *Pseudozyma* sp., *Pichia* sp., *Rhodotorula* sp., *Yarrowia* sp., *Sarocladium* sp., *Cystofilobasidium* sp., *Lachancea* sp., and *Saccharomyces* sp. In the gut tract, several yeast species, such as *L. kluyveri*, *C. macerans*, *R. babjevae*, *R. kratochvilovae*, *R. mucilaginosa*, *S. cerevisiae*, *H. guilliermondii*, *H. opuntiae*, *H. pseudoguillermondi*, *H. uvarum*, *S. roseus*, *S. strictum*, *P. fermentans*, *P. flocculosa*, *P. kluyveri*, and *P. kudriavzevii*, were isolated, but were never found in flowers nor bee products.

In the bee products, a total of 16 yeast genera were identified (7 *Basidiomycetes*, Table 3), of which 4 (*Aureobasidium*, *Starmerella*, *Metschnikowia*, and *Filobasidium*) had multiple species. Most of the species identified in the bee products were not found in the gut mycobiota. The only exceptions are *D. hansenii*, *M. guilliermondii*, *A. pullulans*, *Y. galli*, *C. macerans*, *S. magnoliae*, *F. wieringae*, *S. bombicola*, and *M. pulcherrima*.

### Relative abundance of identified yeasts in all samples

**Fig. 1:**
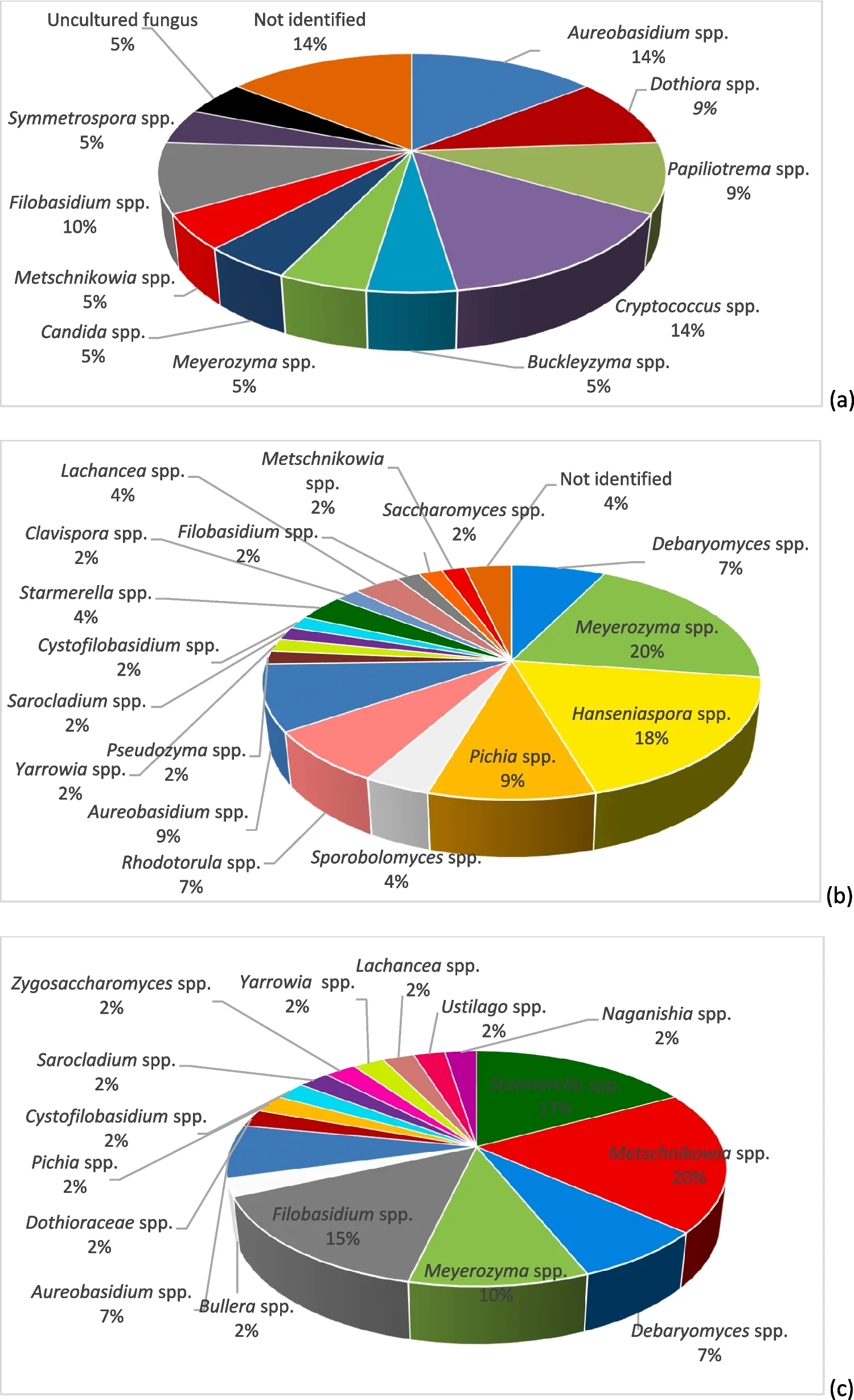
Relative abundance (%) of yeast genera in the three sample typologies analyzed. **a**, **b**, and **c** Graphs showing the occurrence of yeast genera in flowers, the forager bee gut, and bee products, respectively

Figure 1 shows the relative abundance of yeast genera taking into account the origin of isolation. The genera *Aureobasidium* and *Cryptococcus* were the most abundant in flowers, the genera *Meyerozyma* and *Hanseniaspora* were the most abundant in bees, and *Starmerella*, *Metschnikowia*, and *Filobasidium* were the most widely isolated genera in bee products.

A comparative analysis showed that the genera present in flowers, bees, and their products were *Aureobasidium*, *Filobasidium*, *Meyerozyma*, and *Metschnikowia* (Fig. 1a–c). The yeast-like fungus *Aureobasidium* maintained a fairly stable relative abundance in the three ecological niches (14% in flowers, 9% in bee gut, and 7% in products), while the other three genera underwent variations in their relative frequencies.

As expected, the yeasts isolated from bee mycobiota mainly belonged to typical genera with fermenting species and were not detected in flowers. Among these, *Debaryomyces*, *Pichia*, *Yarrowia*, *Starmerella*, *Lachancea*, and the yeast-like *Cystofilobasidium* were also found in bee products. Finally, the genera *Zygosaccharomyces*, *Ustilago*, and *Naganisha* were found only in the bee products.

### Mycobiota and seasonality

**Fig. 2:**
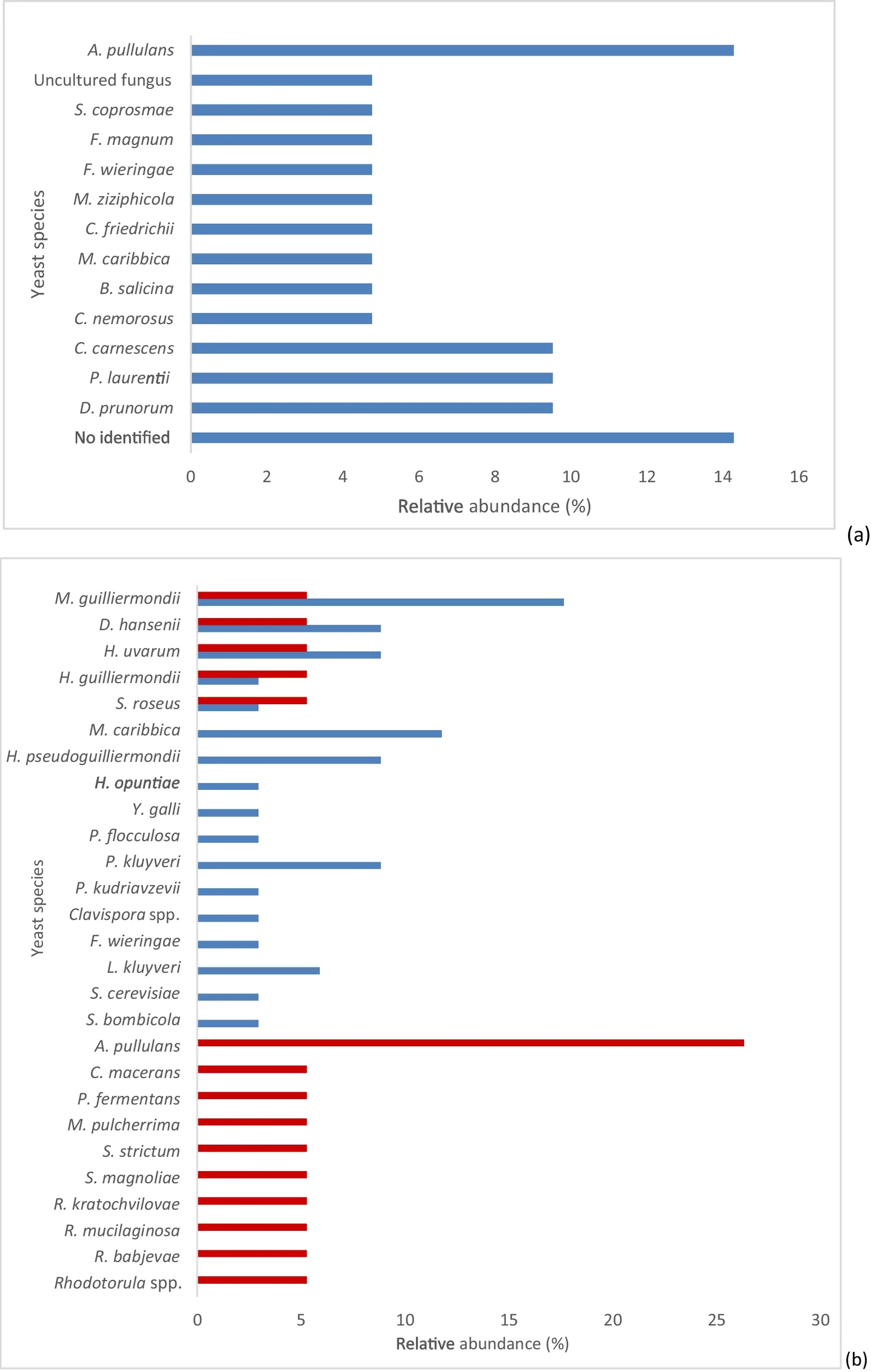

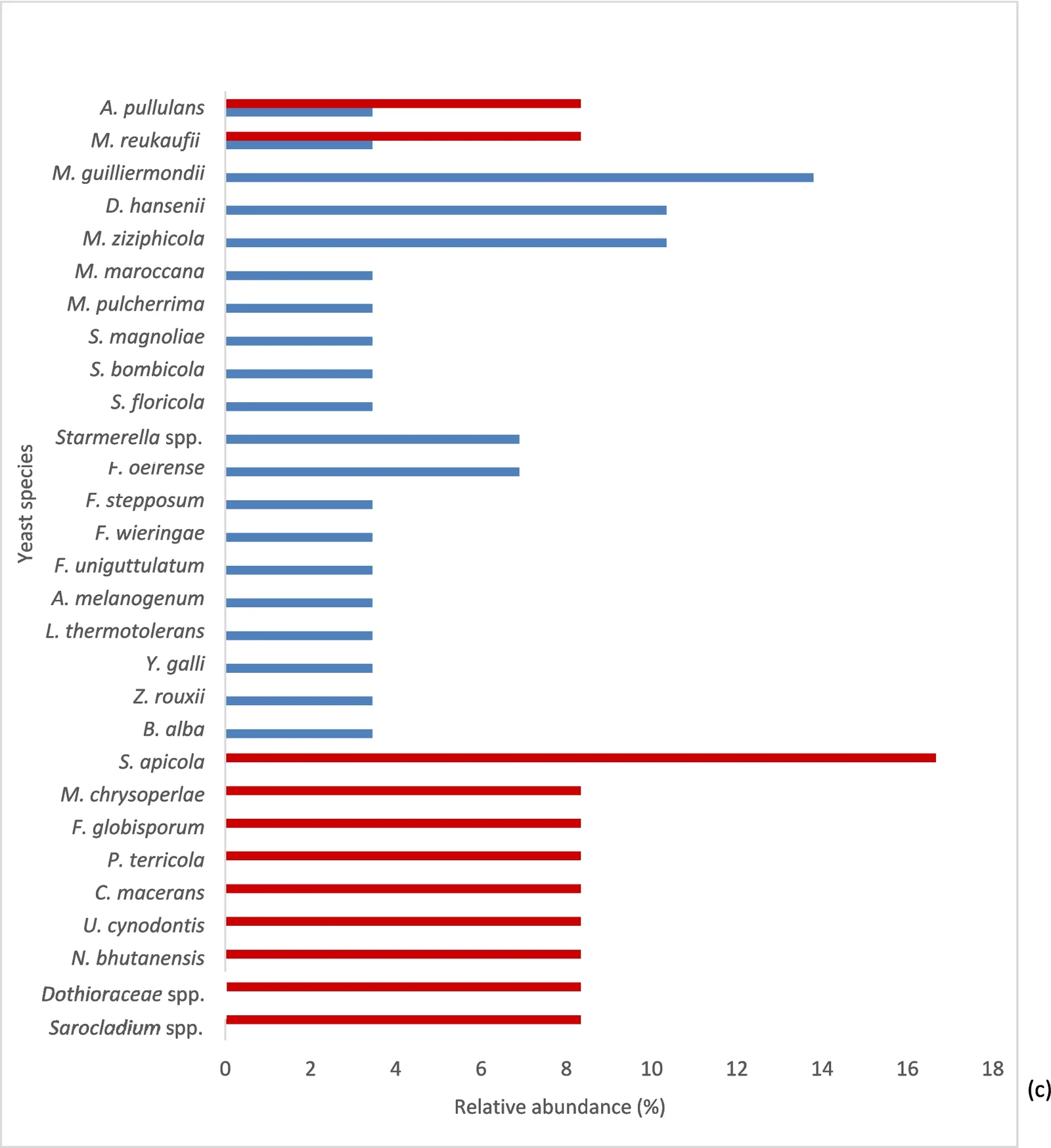
Presence of yeast species, expressed as relative abundance (%), distributed in flower samples (**a**) forager bee gut (**b**) and bee products (**c**) sampled both in summer (  ) and winter (  ), with the exception of flowers, which were available only in summer

Figure 2 shows the relative frequencies of yeasts (classified into genera and species) in the flowers, bee gut, and bee products (Fig. 2a–c) considering seasonality. In this way, it was possible to distinguish the stable yeast populations from those that were sampled from bees inside the hives in the winter months that therefore had no contact with the outside, and those that were sampled during the summer.

From the obtained results, it was possible to split all yeasts of the gut mycobiota of bees into three groups: (i) present only in summer, (ii) present only in winter, (iii) always present. *M. guilliermondii*, *D. hansenii*, *H. uvarum*, *H. guilliermondii*, and *S. roseus* were always present and therefore considered stable components of the gut mycobiota of bees (Fig. 2b).

Analogously, in the bee products collected inside the hives, the yeasts *M. reukafii* and *A. pullulans* were found both in winter and in summer (Fig. 2c); the latter (*A. pullulans*) was among the most abundant in flower samples (Fig. 2a) but also in the gut mycobiota sampled in winter.

Moreover, the yeast distribution reported in Fig. 2 was quantitatively analyzed by the Shannon and diversity (*D*) indices, comparing the diversity of species and considering seasonality, in the different communities: flowers, bee gut, and bee products. The results reported in Table 4 indicate the presence of yeast in each habitat of high population diversity with a high value of the Shannon and diversity indices indicating an abundance of different species in the community.

**Table 4:** Yeast diversity associated with yeast communities in flowers, bee gut and bee products according to seasonality as shown by the Shannon and diversity (*D*) indices

Sample sets	Shannon index	Diversity (*D*)
Flowers	2.54	0.92
Bee gut summer	2.62	0.92
Bee products summer	2.85	0.94
Bee gut winter	2.51	0.90
Bee products winter	2.37	0.91

**Fig. 3:**
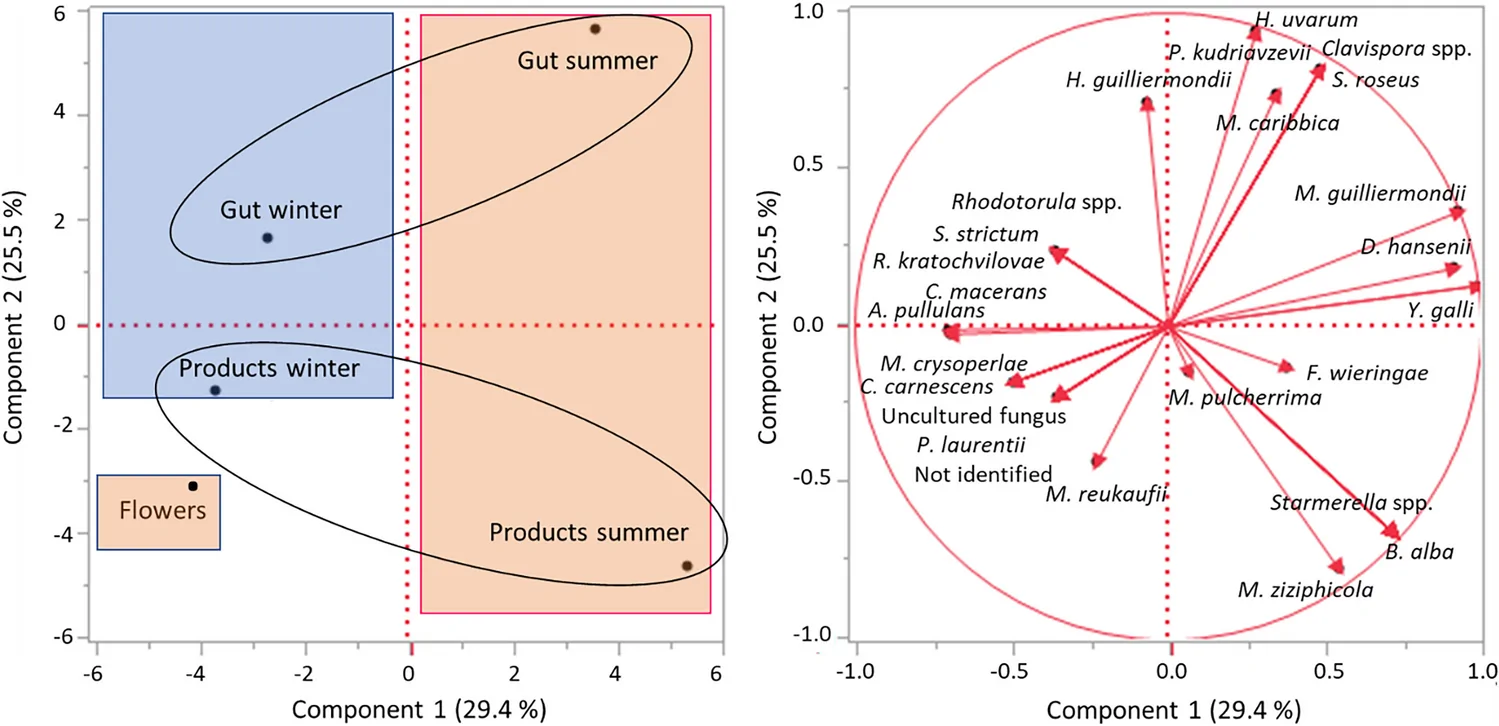
Principal component analysis (PCA) based on the relative abundance of the yeast species detected in each sample typologies (flowers, forager bee gut, and bee products) according to seasonality. Variance explained by PCA (principal component analysis) is Component 1, 29.4% X-axis and Component 2, 25.5% Y-axis

Finally, the relative abundance of all yeast species detected in all samples (flowers, gut, and bee products in summer and winter) was subjected to principal component analysis (PCA), as reported in Fig. 3. The total variance described was 54.9%; PC1 (29.4%) clustered the samples according to seasonality with the summer samples on the right side of the graph and the winter ones on the left side; PC2 (25.5%) subdivided according to the type of sample with the gut samples in the upper part of the graph and the bee’s products in the lower part. The yeast community of flowers was stand-alone and more closely related to winter bee products (lower left quadrant).

## Discussion

Climate change as global warming and precipitation dynamics, specifically extreme rainfall or drought, leads to a strong negative effect on agriculture and the whole ecosystem, and consequently, organisms are threatened (Radchuk et al. 2019). Bioindicators have generated great interest in environmental pollution research; among them, insects are especially useful for assessing the effects of anthropogenic activities on terrestrial ecosystems since they are in close contact with toxic elements present in soil, water, and air (Mullin et al. 2010; Smart et al. 2016). Honeybees are highly susceptible to the effects of climate change both on urban farms and in more peripheral rural areas (Radchuk et al. 2019; Md Meftaul et al., 2020), with considerable negative impacts on their physiological and morphological characteristics (Oskay 2021).

Similarly, knowledge of the gut microbiota composition of honeybees allows us to better understand their state of health and interactions with the surrounding environment. For this, several studies on the intestinal microbiota of insects have become of great interest as measures for ecosystem health (Mereghetti et al. 2017; Munoz-Benavent et al. 2021). For bees, it is well understood that their gut microbiota composition is mainly influenced by diet, and dysbiosis makes bees more susceptible to disease, shortens their lifespan, and is the main cause of the collapse of colonies. In the last decade, EU-funded projects helping to protect bees across Europe have increased (European Research Executive Agency 2022). Major progress has been made in defining some of the dominant bacterial members of the microbial community and in identifying their roles in gut metabolism (Tauber et al. 2019); however, the fungal population remains poorly understood, despite yeasts being actively involved in the fermentative process that converts pollen into bee bread, a fundamental product for the development of bee colonies (Gilliam 1979; Detry et al. 2020).

In the present study, a harvesting campaign of culturable yeasts isolated from the gut of honeybees, their foraging environment (flowers), and related bee products was carried out to understand bee gut mycobiota composition and contribute to research on bee health as an urgent matter for their protection. The distribution, diversity, frequency, and seasonality of yeast mycobiota were investigated after molecular identification.

The results show how although the abundance of yeasts is significantly similar among the samples analyzed, there is great variability in the genera and species found. Similar high variability was also described by Callegari et al. (2021) regarding the abundance of culturable fungi associated with different areas of the gut.

Further evaluation of the obtained results highlighted that in flowers, there were more yeasts than aerobic bacteria, as confirmed by several naturalistic studies. Indeed, some investigations on yeasts in flower nectar have shown strict yeast-bacteria and yeast-yeast competition (Tucker and Fukami 2014; Toju et al. 2018). The presence of yeasts in flowers seems to be involved in attracting insects through the production of volatile extracellular substances (generally VOCs) that act as traps for pollinating insects, mainly honeybees (Rering et al., 2017; Pozo et al. 2020).

*Aureobasidium*, together with *Meyerozyma*, *Pichia*, and *Hanseniaspora*, are also the main residents of the gut tract. These last genera were also found by Callegari et al. (2021), although in different proportions. The cultivation-based strategy may differ from Illumina or NGS (Next Generation Sequencing) methodology and diversity and relative abundance may be influenced by viability and techniques. The presence of *Starmerella* in both the gut and bee products is consistent with other results, which demonstrated that the *Starmerella* genus is predominately associated with stingless bees (Teixeira et al., 2023; da Costa Neto and Morais, 2020) .

The genera *Metschnikowia*, *Meyerozyma*, and *Filobasidium* were the most abundant in bee products, and these results are partially in accordance with results, which described *Metschnikowia* and *Zygosaccharomyces* (found only at low concentrations in the products sampled in this study) as the most abundant genera in bee bread and pollen (Detry et al. 2020). *Starmerella*, *Metschnikowia*, *Meyerozyma*, *Pichia*, and *Debaryomyces* were found both in the bee gut tract and in bee products as fermentative yeasts with active roles in the pollen fermentation process for the production of bee bread (Didaras et al. 2020; Mohammad et al. 2021).

Exploring the relationships existing between the identified yeasts and the various sources of isolation clearly emphasized that yeasts belonging to *Aureobasidium*, *Filobasidium*, *Meyerozyma*, and *Metschnikowia* were present among flowers, bee guts, and bee products, probably highlighting a continuous flux from the environment to bees and vice versa. Moreover, considering the dynamics of recovered yeasts in relationship with seasonality, it is possible to classify the population into stable and transient; *M. guilliermondii*, *D. hansenii*, *H. uvarum*, *H. guilliermondii*, and *S. roseus* were present both in summer and winter; therefore, they could be considered stable components of the gut mycobiota of bees. Similarly, the yeasts *M. reukafii* and *A. pullulans* were present in both bee products collected in winter and in summer. Most likely, some of these environmental yeasts, over the seasons, have developed adaptive mechanisms to establish a symbiotic relationship with the host. In our results, the genus *Filobasidium* appeared, although present in all collected samples, to be a nonstable colonizer. This is in line with what was reported by Klaps et al. (2020), who described the genus *Filobasidium* as being most associated with momentary transit within the gastrointestinal tract of the bee without establishing any stable colonization.

*A. pullulans* was the most abundant yeast species detected in flowers and only in bee guts sampled in winter. Its absence in gut samples collected in summer could be due to a change in nutrient availability and bee metabolism during their active period (summer). The relative abundance of hexoses in the gut tract could be more favorable for osmophilic and fermenting species that dominated in summer. Indeed, a wide occurrence of fermenting and osmophilic yeast species was found only in the summer period in both bee gut and bee product ecological niches. However, the persistence of this yeast in all these ecological samples denotes its competition toward the other yeast species colonizing bee-related environments. Hence, there is a concept of natural filtering where the environment functions as a sieve through which species with unsuited traits will be ride off from local communities if not able to develop adaptative characteristics (Kraft et al. 2015). The use of two specific statistical biodiversity indicators revealed lower yeast diversity in winter than in summer, with both the Shannon and diversity indices, in line with the fact that in this period, the bees have no exchanges with the outside world.

This could also be related to the climate conditions occurring during the sampling process: 2021 was characterized by a relatively warm winter followed by a late frost in May. June and July were characterized by an absence of rain, as also reported by the SPI-12 index, relating to the Marche region (source ASSAM report 2021). Under these conditions, bees have not imported enough nectar and pollen, affecting the population composition of the bee gut and related surrounding environment and bee products.

By studying the dynamics of the gut mycobiome could possibly understand the relationships between honeybees and environments, through their products, also highlighting the processes that govern the colonization of specialized gut communities as well as the ways through which gut communities affect (Zheng et al. 2018) honeybees and related bee products. This knowledge could also be crucial for explaining and predicting the degree of host resilience during changes in environmental conditions and factors of stress, such as pathogens. Moreover, these findings—which could be repeated in the coming years—can be helpful for new studies, with the possibility of enriching virtuous pollen products (such as superfoods) fortified with beneficial microorganisms or probiotics that could increase the nutritional value, further enhancing human health.

## Supplementary Information

Below is the link to the electronic supplementary material.

Supplementary file1 (PDF 173 kb)

## Data Availability

All data generated or analyzed during this study are included in this published article (and its supplementary information files). DNA sequences have been deposited in the NCBI GenBank database under the project titled Sequence from Bees (SUB12617947) with sequence accession numbers OQ357433 to OQ357537.
