# Supplementary material for: Yeast communities related to honeybees: occurrence and distribution in flowers, gut mycobiota, and bee products
Source: Appl Microbiol Biotechnol. 2024 Jan 26;108(1):175. doi: 10.1007/s00253-023-12942-1 (PMC10817854; doi:10.1007/s00253-023-12942-1)
Supplement: Supplementary file 1 — Supplementary file1 (PDF 173 kb) [file 253_2023_12942_MOESM1_ESM.pdf]

Journal name: Applied Microbiology and biotechnology
Manuscript Title: Yeast communities related to honeybees: occurrence and distribution in flowers, gut mycobiota and bee products. (AMAB-D-23-01808R1)

The name(s) of the author(s): Alice Agarbati, Silvia Gattucci, Laura Canonico, Maurizio Ciani, Francesca Comitini

The affiliation(s) and address(es) of the author(s): Department of Life and Environmental Sciences, Polytechnic University of Marche, Via Brecce Bianche, 60131 Ancona, Italy.

The e-mail address, telephone and fax numbers of the corresponding author: f.comitini@univpm.it, +39 071.2204150

Supplemental Material

Table S1. Presence of yeasts, mould and aerobic bacteria in all samples. The microbial population was detected by viable cell counts and the results are expressed as Log CFU/flower; Log CFU/gut and Log CFU/g of product for bee food supply, bee gut tract and bee products.

		Log CFU		
**Source**	**Code of sample**	**Yeasts**	**Mould**	**Aerobic bacteria**
i) Bee food supply (flowers)	**1C**	5.53	3.30	2.69
	**2C**	0.00	5.32	2.90
	**3C**	4.95	2.00	1.72
	**4C**	4.48	2.00	3.10
	**5C**	1.10	2.00	4.26
	**6**	5.00	3.70	0.00
	**7**	3.90	3.00	1.78
	**8**	1.46	3.30	0.00
	**9**	0.00	0.00	0.00
	**10**	5.08	0.00	0.00
	**11**	6.25	0.00	0.00
	**12**	4.89	0.00	0.00
	**13**	5.38	0.00	0.00
	**1 M**	1.00	4.08	0.57
	**2 M**	1.29	3.60	1.74
	**3 M**	0.00	3.90	2.32
	**8C**	1.09	3.30	0.00
	**1F**	5.84	4.60	2.97
	**2F**	0.00	5.00	2.49
	**3F**	0.90	4.08	2.90
	**14**	6.54	5.70	6.08
	**15**	6.52	5.70	3.32

		**16**	0.98	3.60	0.00
		**17**	5.15	3.30	3.48
**ii) Forager bee**	**Hive C**	**i C1**	2.00	0.00	2.00
		**i C2**	3.48	0.00	1.22
		**i C3**	3.78	0.00	1.50
		**i C4**	6.00	0.00	0.90
		**i C5**	3.68	0.00	2.00
		**i C6**	4.48	0.00	0.20
		**i C7**	2.00	0.00	2.80
		**i C8**	4.10	0.00	0.00
		**i C9**	4.70	0.00	3.58
		**i C10**	5.30	0.00	3.55
		**p C1**	0.90	0.00	0.00
		**p C2**	1.00	0.00	0.00
		**p C3**	0.90	1.11	0.00
	**Hive M**	**i M1**	2.48	2.00	0.00
		**i M2**	3.48	0.00	4.08
		**i M3**	3.54	0.00	2.10
		**i M4**	3.79	0.00	1.90
		**i M5**	4.00	0.00	3.85
		**i M6**	3.90	0.00	3.92
		**i M7**	3.30	0.00	2.75
		**p M1**	1.08	2.49	0.00
		**p M2**	2.70	2.01	0.00
		**p M3**	1.48	1.82	0.00
		**p M4**	0.00	0.00	1.11
	**Hive U**	**i U1**	4.10	0.00	5.00
		**i U2**	3.70	0.00	5.00
		**i U3**	3.81	0.00	3.00
		**i U4**	3.91	0.00	0.00
		**i U5**	3.92	0.00	2.20
		**i U6**	3.11	0.00	2.90
		**i U7**	2.36	0.00	1.00
		**i U8**	2.00	0.00	2.50
		**i U9**	2.60	2.00	4.67
		**i U10**	5.85	0.00	0.00
		**i U11**	4.10	0.00	3.35
		**p U1**	3.30	0.00	1.18
		**p U2**	3.48	1.38	0.00
		**p U3**	3.70	0.00	0.00
		**p U4**	4.30	0.00	0.60
**iii) Bee products**	**Hive C**	**b C1**	0.00	3.78	4.94
		**b C2**	3.62	0.00	0.00

	**b C3**	5.36	0.00	0.00
	**b C4**	3.00	3.70	4.73
	**b C5**	5.60	4.36	4.52
	**b C6**	3.00	3.30	0.00
	**pr C1**	4.00	0.00	0.00
	**pr C2**	3.60	0.00	0.00
	**pr C3**	6.48	0.00	0.00
	**po C1**	0.00	3.48	0.00
**Hive M**	**b M1**	3.85	0.00	0.00
	**b M2**	3.34	0.00	0.00
	**pr M1**	5.92	0.00	0.00
	**pr M2**	6.96	0.00	0.00
	**b M3**	3.48	3.30	4.81
	**b M4**	0.00	3.70	4.36
	**pr M3**	0.00	3.30	4.68
	**po M1**	4.60	3.48	3.95
**Hive U**	**b U1**	0.00	0.00	0.00
	**b U2**	5.63	0.00	0.00
	**b U3**	3.60	0.00	0.00
	**b U4**	7.26	0.00	0.00
	**b U5**	5.68	0.00	0.00
	**b U6**	3.78	6.28	5.00
	**b U7**	3.70	4.00	5.18
	**b U8**	2.00	0.00	0.00
	**pr U1**	0.00	3.00	4.04
	**po U1**	4.60	3.48	4.57
	**po U2**	5.60	0.00	4.30
	**po U3**	6.45	4.00	0.00
